# HLA-A2-Restricted Epitopes Identified from MTA1 Could Elicit Antigen-Specific Cytotoxic T Lymphocyte Response

**DOI:** 10.1155/2018/2942679

**Published:** 2018-11-25

**Authors:** Yahong Wu, Wenjie Zhai, Xiuman Zhou, Zhiwei Wang, Yan Lin, Ling Ran, Yuanming Qi, Yanfeng Gao

**Affiliations:** School of Life Sciences, Zhengzhou University, Zhengzhou 450001, China

## Abstract

Overexpression of metastasis-associated protein 1 (MTA1) has been observed in many human malignancies and is significantly related to tumor invasion and metastasis, therapeutic resistance to radiation and chemotherapy, making MTA1 an ideal candidate tumor antigen. We identified several human leukocyte antigen- (HLA-) A2-restricted epitopes in MTA1 and evaluated their binding ability to HLA-A^∗^0201 molecules. Subsequently, a recombinant fragment encompassing the dominant epitopes, MTA1_(1–283)_, was expressed, and the abilities of the selected epitopes of MTA1 and the MTA1_(1–283)_ fragment to induce cytotoxic T lymphocytes (CTLs) were examined. Our results indicated that the epitopes and MTA1_(1–283)_ fragment elicited HLA-A2-restricted and antigen-specific CTL responses both *in vitro* and *in vivo*. The new epitopes identified here may help promote the development of new therapeutic vaccines for HLA-A2^+^ patients expressing MTA1.

## 1. Introduction

Over the past decade, tumor immunotherapy has shown its unique advantages and good clinical efficacy in the treatment of malignant tumors, and numerous immune checkpoint inhibitors that target cytotoxic T lymphocyte-associated protein 4 (CTLA-4) and programmed cell death 1 (PD-1)/programmed death-ligand 1 (PD-L1) including ipilimumab [1], nivolumab, and pembrolizumab [2] have been approved by the Food and Drug Administration (FDA). The clinical efficacy of immune checkpoint inhibitors depends on the presence of tumor-specific CD8^+^ cytotoxic T lymphocytes (CTLs) in cancer patients, and the basic principle is to utilize the patient's own CTLs [3]. Thus, activating tumor-specific T lymphocytes to recognize the epitopes processed from antigens and presented on the tumor cell surface may act synergistically with immune checkpoint inhibitors in tumor treatment [4].

Antigens related to oncogenic processes are promising immunotherapy targets because of their lower likelihood of being downregulated to allow tumor immune escape. Metastasis-associated protein 1 (MTA1), containing 730 amino acids, was identified by Toh et al. in 1994 and is related to tumor metastasis [5]. The AP1/MTA1/Pol II complex is involved in activating oncogene expression [6], whereas the MTA1/HDAC2 complex functionally inhibits the expression of tumor suppressor genes, eventually leading to the development of malignancy [7, 8]. Overexpression of MTA1 has been observed in many tumors, such as breast [9], colorectal [10], gastric [11], and esophageal [12] cancers. In addition to its involvement in the invasion and metastasis, MTA1 has been implicated in proliferation, angiogenesis, and therapeutic resistance to radiation and chemotherapy, which can eventually lead to tumor recurrence [13, 14]. Our previous study found that MTA1 was upregulated in tumor cells treated with chemotherapy drugs such as cisplatin, 5-fluorouracil, and pacitaxel. Therefore, MTA1 has been considered an ideal target for cancer immunotherapy [15]. However, the immune-dominant CTL epitopes of MTA1 have not been identified.

Here, we predicted human leukocyte antigen- (HLA-) A2-restricted candidate peptides of MTA1 using the online program NetCTL1.2 (http://www.cbs.dtu.dk/services/NetCTL/), which is commonly used to predict potential CTL epitopes [16]. Candidate peptides were synthesized, purified, and subjected to a T2 cell-binding assay to determine their binding affinity to HLA-A^∗^0201 molecules. Subsequently, we assessed the ability of the selected peptides to elicit specific immune responses both *in vitro* (using peripheral blood mononuclear cells (PBMCs) from HLA-A^∗^02^+^ healthy donors) and *in vivo* (using HLA-A2.1/K^b^ transgenic mice) using an interferon gamma (IFN-*γ*) secretion assay and lactate dehydrogenase (LDH) cytotoxicity assay. Based on the antigen epitope prediction and immune response results, a recombinant MTA1_(1–283)_ fragment containing the predominant epitopes of MTA1 was expressed, and we examined the ability of the MTA1_(1–283)_ fragment to induce CTLs both *in vitro* and *in vivo*.

## 2. Materials and Methods

### 2.1. Cell Culture, Human Blood Samples, and Animals

The lymphoblastic cell line T2 (TAP-deficient, HLA-A^∗^0201 overexpressed) used in the binding assay was kindly gifted by Professor Yuzhang Wu (Third Military Medical University, China). The HLA typing of tumor and normal cells was performed by Beijing Santa Wagon Biotechnologies Co. LTD. The human normal esophageal epithelial cell Het-1A (HLA-A2^+^, MTA1^low^), human primary umbilical vein endothelial cell HUVEC (HLA-A2^+^, MTA1^low^), human breast cancer cell lines MDA-MB-231 (HLA-A2^+^, MTA1^+^) and MCF-7 (HLA-A2^+^, MTA1^+^), and human colorectal cancer cell lines SW620 (HLA-A2^+^, MTA1^+^), SW480 (HLA-A2^+^, MTA1^+^), and HT-29 (HLA-A2^−^, MTA1^+^) were all purchased from American Type Culture Collection (ATCC, Rockville, MD, USA). All cells were cultured in RPMI 1640 medium (Invitrogen, Grand Island, USA) at 37°C in a humidified atmosphere of 5% CO_2_ and 95% air, supplemented with 10% fetal bovine serum (FBS, BI, Israel), 2 mM L-glutamine, 100 units/ml penicillin, and 100 *μ*g/ml streptomycin.

We use flow cytometry to select HLA-A^∗^02^+^ healthy donors (anti-human HLA-A2 PE-Cyanine7 mAb, BB7.2 (eBioscience, USA) was used), then 40 ml human peripheral blood samples were isolated from eight HLA-A^∗^02^+^ healthy donors, verbal informed consent was obtained from all individuals, and the collection was approved by the Ethics Committee of Zhengzhou University. Experiment was performed using protocols approved by the Ethics Committee of Zhengzhou University [17].

HLA-A2.1/K^b^ transgenic mice were purchased from Model Animal Research Center of Nanjing University (Nanjing, China). Then, mice were group-housed and maintained in specific pathogen-free facilities (SPF) and fed SPF food and autoclaved water in our laboratory. Mice at six- to eight-week-old were randomly divided into five mice per group. All procedures described in the experiment were conducted under the approval of Institutional Animal Care and Use Committees of Zhengzhou University.

### 2.2. Peptides

Computational prediction of MTA1-derived HLA-A2-restricted CTL epitopes was conducted using online T cell epitope prediction program NetCTL1.2 [18]. Candidate peptides were synthesized using the Fmoc solid-phase strategy and then purified with the purity of >95% using reverse phase high-performance liquid chromatography (RP-HPLC) and analyzed by electrospray ionization mass spectrometry (ESI–MS). In the T2 binding assay [17], cyclooxygenase-2 (COX-2)_321–329_ (ILIGETIKI) and HBcAg_18–27_ (FLPSDFFPSV) were used as positive controls. In the *in vivo* assay [19], IA^b^-restricted HBV cores antigen-derived T helper epitope (sequence 128–140: TPPAYRPPNAPIL) was used. Purified peptides were dissolved in DMSO (10 mg/ml) and stored at −80°C.

### 2.3. Analysis of MTA1 Expression by Western Blot

The protein expression of MTA1 (81 kDa) was evaluated by Western blot [20, 21]. In brief, the cells were collected and lysed using RIPA buffer (Applygen, China); the protein concentrations of the resulting lysates were normalized, and 30 *μ*g protein from each cell was added to different lanes of the SDS-PAGE gel (10%) and transferred to the PVDF membrane (Bio-Rad, USA). Membranes were blocked with 5% skim milk in TBST buffer (50 mM Tris–HCl, 0.1% Tween-20, pH 7.4) for about 1.5 h. Then the membranes were washed three times for 5 min with TBST. Further, the membranes were incubated overnight at 4°C with the indicated primary antibody—rabbit anti-MTA1 antibody (cat: bs-1412R, dilutions: 1 : 1500, Bioss Antibodies, China/cat: 5646, dilutions: 1 : 500, Cell Signaling Technology, USA) or rabbit polyclonal anti-*β*-actin antibody (cat: bs-0061R, dilutions: 1 : 50,000, Bioss Antibodies, China)—and then washed three times in TBST. Finally, corresponding secondary antibodies for MTA1 and *β*-actin were further incubated, and the bands were visualized with the ECL plus system. Then, gray analysis of the MTA1 protein band intensities (relative to the *β*-actin band intensity) was carried out by Image J.

### 2.4. T2 Binding and Stabilization Assays

The binding affinity of these peptides to HLA-A^∗^0201 was evaluated according to our previous study [17, 20, 22]. Briefly, T2 cells (1 × 10^6^ cells/ml) were incubated with each peptide (50 *μ*g/ml) and 3 *μ*g/ml human *β*2-microglobulin (*β*2-M, Merck, Germany) in serum-free RPMI 1640 medium for 18 h at 37°C in a humidified atmosphere of 5% CO_2_. Then cells were washed twice and incubated with the anti-human HLA-A2 PE-Cyanine7 mAb, BB7.2 (eBioscience, USA) for 0.5 h at 4°C. Finally, cells were collected, washed, and analyzed using flow cytometry (FACSCalibur, Becton Dickinson, USA). The fluorescence index (FI) calculation was performed as follows: FI = [(mean fluorescence intensity (MFI) of the given peptide − background) − (MFI of the PBS control group − background)]/[(MFI of the PBS control group − background)]. The MFI value of cells untreated with peptides and antibodies was employed as background. Peptides with an FI factor of more than 1.5 were classed as high-affinity candidates [22].

For the stability assay [23, 24], each of the selected peptides (50 *μ*g/ml) was incubated with T2 cells (1 × 10^6^ cells/ml) for 18 h in serum-free RPMI 1640 medium supplemented with 3 *μ*g/ml *β*2-M at 37°C. Thereafter, cells were washed twice and incubated with brefeldin A (10 *μ*g/ml, eBiosciences, USA) for 1 h. Subsequently, the cells were washed and incubated for 0, 2, 4, and 6 h at 37°C. Cells were washed twice, stained with BB7.2 for 0.5 h at 4°C. Finally, cells were collected, washed, and analyzed by flow cytometry. Dissociation complex 50 (DC_50_) was defined as time (*t*) required for the loss of 50% of the HLA-A^∗^0201/peptide complexes stabilized at 0 h [25].

### 2.5. Vector Construction, Protein Expression, and Purification

Based on antigen epitope prediction and immune response results, we found that the predominant epitopes of MTA1 were concentrated on the first 283 amino acids. The sequence of the MTA1_(1–283)_ fragment was spliced by polymerase chain reaction (PCR). The primers used were sense 5′-CGGAATTCATGGCCGCCAACATGTAC-3′ (EcoR I restriction site underlined) and antisense 5′-ATAAAGCTTTCACTCGTCCCTGCAGAGC-3′ (Hind III restriction site underlined). The purified products were cloned into the pET28a expression vector which had been digested with EcoR I/Hind III restriction enzymes to generate pET28a-MTA1_(1–283)_ [26, 27]. The host *E. coli* Transetta (DE3) cells were transformed with constructed recombinant vectors, harvested and then lysed by sonication [28, 29]. After centrifugation (12,000 rpm, 20 min), expressed proteins as inclusion bodies were solubilized and purified by Ni-NTA Sefinose™ Resin column (Shanghai Sangon Biotech, China). Then the protein was dialyzed to refolding. The protein was dissolved into PBS (pH 7.4) at a final concentration of 2 mg/ml.

### 2.6. *In Vitro* CTL Induction

CTL induction *in vitro* was conducted according to the procedures previously described [17, 30]. Briefly, PBMCs from eight HLA-A^∗^02^+^ healthy donors were isolated using Ficoll-Paque density gradient centrifugation and resuspended in RPMI 1640 medium supplemented with 10% FBS. Then, PBMCs were stimulated three times (once a week) with the indicated peptides (10 *μ*g/ml) or protein MTA1_(1–283)_ (at final concentrations of 0, 1, 10, 30, and 50 *μ*g/ml) for 21 days. Human recombinant cytokine interleukin-2 (IL-2) (50 U/ml) was added on day 3 and 1 day after each stimulation. The cytotoxic assay (lactate dehydrogenase (LDH) assay) and IFN-*γ* secretion enzyme-linked immunospot (ELISPOT) assay were performed on day 21.

### 2.7. *In Vivo* CTL Induction

*In vivo* induction of peptide-specific CTLs was performed as previously described [17, 30]. Briefly, peptide (100 *μ*g) and the IA^b^-restricted HBV-core_128_ T helper epitope (140 *μ*g) emulsified in incomplete Freund's adjuvant (IFA) were used to subcutaneously immunize at the base of the tail of HLA-A2.1/K^b^ transgenic mice three times on days 0, 5, and 10. On day 11, all mice were sacrificed through cervical dislocation. Spleen lymphocytes were isolated and restimulated once with 10 *μ*g/ml peptide and 10 U/ml mIL-2 *in vitro* for an additional 6 days. LDH and IFN-*γ* ELISPOT assays were performed on day 6 after *in vitro* stimulation. E/T ratios used in the LDH assay were 20 : 1, 40 : 1, and 80 : 1.

To test the effects of protein, each HLA-A2.1/K^b^ transgenic mouse was injected subcutaneously at the base of the tail with 100 *μ*g MTA1_(1–283)_ or the same volume of PBS emulsified in IFA twice on days 0 and 7. On day 14, spleen lymphocytes were harvested and stimulated by peptide pool (10 *μ*g/ml) *in vitro* for another 6 days. LDH, IFN-*γ* ELISPOT, and IFN-*γ* intracellular cytokine staining assays were performed on day 6 after the *in vitro* stimulation. E/T ratios used in the LDH assay were 12.5 : 1, 25 : 1, and 50 : 1.

### 2.8. Enzyme-Linked Immunospot (ELISPOT) Assay

The ELISPOT assay was performed using a commercial kit (Dakewe, China) according to the procedures previously described [29]. Briefly, T2 cells incubated with/without candidate peptide (50 *μ*g/ml) were used as the stimulating cells, effector cells (1 × 10^5^) and stimulator cells (peptide-pulsed T2 cells or T2 cells only, 1 × 10^5^) were seeded into the 96-well plate precoated with an anti-human (or anti-mouse) IFN-*γ* antibody. After incubation at 37°C for 18 h, cells were removed, and plates were processed for analysis as instructed. The spot number was determined automatically using a computer-assisted spot analyzer (Dakewe, China).

### 2.9. Cytotoxicity Assay

Cytotoxic activity of induced T cells was tested based on the quantitation of LDH release using the nonradioactive cytotoxicity assay kit (Promega, USA) at the gradient E/T ratio [29, 31]. Target cells which expressed different levels of MTA1 or peptide-pulsed T2 cells were used as target cells. The anti-human HLA-A2 antibody (BB7.2) was added 30 min before adding effector cells to determine the HLA restriction. Target cells (5 × 10^3^/well) were cocultured with the effector cells at 37°C for 4 h. The percentage of specific lysis of the target cells was determined using the equation: percentage of specific lysis = [(experimental release − effector spontaneous release − target spontaneous release)/(target maximum release − target spontaneous release)] × 100.

### 2.10. IFN-*γ* Intracellular Cytokine Staining Assay

To test the protein MTA1_(1–283)_ stimulation of CTL response *in vivo*, IFN-*γ* release of induced CTLs was determined by the intracellular cytokine staining assay according to our former study [32]. In brief, on day 14, spleen lymphocytes were harvested and stimulated by peptide pool (10 *μ*g/ml) *in vitro* for another 6 days. Then, restimulated splenocytes were incubated with peptide-loaded T2 cells or T2 cells only for 3 h. Subsequently, protein transport inhibitor cocktail was added to splenocytes for another 4 h. Then, those cells were stained with Anti-mouse CD3 PerCP-eFluor® 710 (clone: 17A2, eBioscience) and Anti-mouse CD8*α* PE (clone: 53–6.7, eBioscience) at 4°C for 30 min. And those cells were fixed by IC fixation buffer (cat no: 00-8222, eBioscience) at room temperature for another 30 min; then those cells were washed twice by permeabilization buffer (cat no: 00-8333-56, eBioscience). After, those cells were incubated with Anti-mouse IFN-*γ* APC (clone: XMG1.2, eBioscience) at 4°C for 30 min. Those cells were washed twice by permeabilization buffer and analyzed by flow cytometry.

### 2.11. Statistical Analysis

All data represent means ± SD. Statistical comparisons were analyzed by one-way analysis of variance (ANOVA) [17]. ^∗^ *P* < 0.05, ^∗∗^ *P* < 0.01, and ^∗∗∗^ *P* < 0.001 were considered statistically significant vs. the control group.

## 3. Results

### 3.1. MTA1 Protein Expression in Target Cells

**Figure 1:**
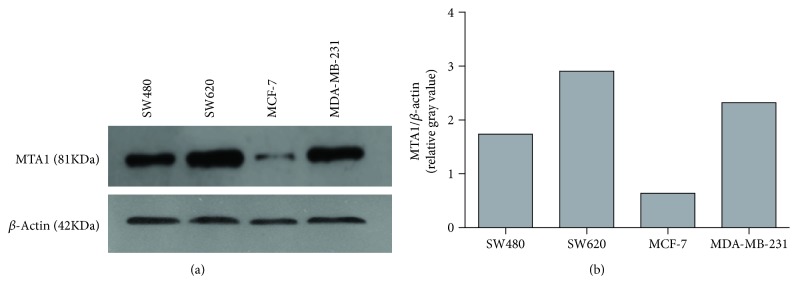
Protein expression of MTA1 by Western Blot analysis. The protein expression of MTA1 (81 kDa) was evaluated in four human cancer cell lines. *β*-Actin (42 kDa) was used as loading control, and rabbit polyclonal anti-MTA1 antibody (cat: bs-1412R, dilutions: 1 : 1500, Bioss Antibodies, China) or rabbit polyclonal anti-*β*-actin antibody (cat: bs-0061R, dilutions: 1 : 50,000, Bioss Antibodies, China) was used in this assay. (a) The bands representing MTA1 and *β*-actin in each cancer cell line. (b) Gray analysis of the MTA1 protein band intensities (relative to the *β*-actin band intensity) was carried out by Image J.

The protein expression of MTA1 was evaluated by Western blot analysis (Figure 1). MTA1 protein expression was higher in SW620 than in SW480 cells and higher in MDA-MB-231 cells than in MCF-7 cells. Although the SW620 and SW480 cell lines originated from the same patient, SW620 cells were derived from a metastatic lesion in the patient, and the metastatic activity is higher in MDA-MB-231 cells than in MCF-7 cells, consistent with the higher MTA1 expression in SW620 and MDA-MB-231 cells [9]. Our results support that MTA1 is positively correlated with tumor metastasis. In addition, we also evaluated the expression of MTA1 in two normal cell lines, HUVEC and Het-1A cells, using another MTA1 antibody (band in HUVECs can be visualized when the dilution of the MTA1 antibody increased to 1 : 500, cat: 5646, CST) and found significantly lower expression of MTA1 in both cell lines compared with MCF-7 cells (Figure S1). Thus, because they express variable levels of MTA1, we selected MDA-MB-231, MCF-7, HUVEC, Het-1A, and SW620 cells as target cells in subsequent cytotoxicity experiments.

### 3.2. Epitope Prediction and Synthesis

As shown in Table 1, the top five scoring peptides predicted in the NetCTL1.2 database (P22, P57, P109, P129, and P173) were preferentially selected and synthesized. The ESI–MS results suggested that all five peptides were synthesized correctly.

### 3.3. Binding and Stability Assays

The binding affinity of these peptides and the stability of the peptide/HLA-A^∗^0201 complexes were evaluated using HLA-A^∗^0201-overexpressing T2 cells according to our previous method [17]. As shown in Table 1, P109, P22, and P57 showed high binding affinity to the HLA-A^∗^0201 molecule (FI values of 2.61, 2.08, and 1.56, respectively), P129 showed moderate affinity (FI value of 1.28), and P173 showed the lowest affinity (FI value of 0.22). In the stability assay, P109, P22, P57, and P129 formed different stable peptide/HLA-A^∗^0201 complexes (DC_50_ > 6 h, >2 h, >6 h, and >6 h, respectively). Based on these results, P22, P57, P109, and P129 were selected to evaluate their ability to induce T cell responses *in vitro*.

### 3.4. *In Vitro* IFN-*γ* Secretion from Peptide-Specific CTLs

**Figure 2 fig2:**
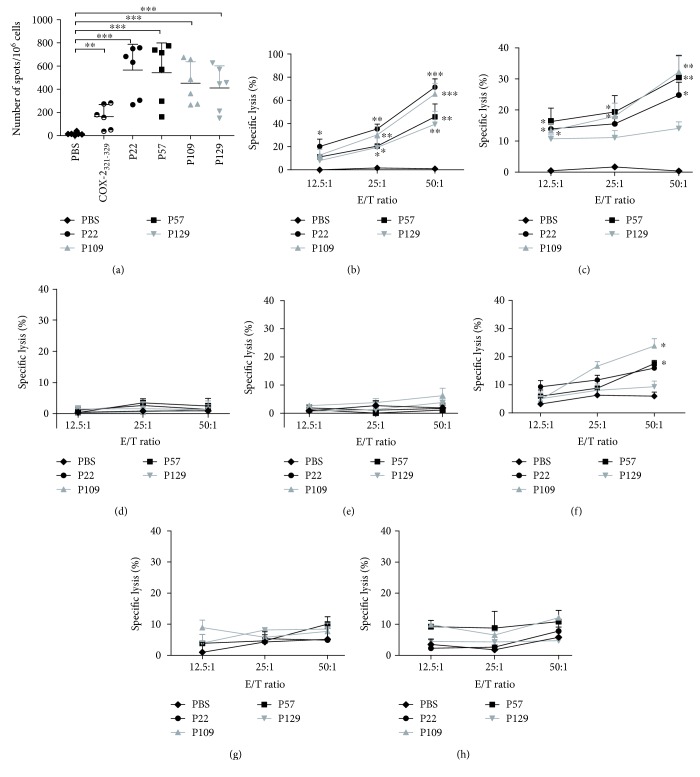
IFN-*γ* release and lyse target cell ability of peptide-induced CTLs. PBMCs from six HLA-A^∗^02^+^ healthy donors were collected and induced by the indicated peptides (10 *μ*g/ml) three times in RPMI 1640 medium supplemented with 3 *μ*g/ml *β*2-M (once at each stimulation), interleukin-2 (50 U/ml, on day 3 and 1 day after each stimulation), and 10% FCS for 21 days. CTLs induced by COX-2_321–329_ were used as positive controls. On day 21, the CTLs were collected, and the IFN-*γ* secretion and cytotoxic activity of the CTLs were measured. (a) CTL secretion of IFN-*γ* was detected by the ELISPOT assay (*n* = 6). T2 cells loaded with/without peptide (50 *μ*g/ml) for 4 h were used as the stimulating cells. LDH cytotoxicity assays were performed (*n* = 3) using the following target cell lines: (b) SW620 (HLA-A2^+^, MTA1^+^), (c) MDA-MB-231 (HLA-A2^+^, MTA1^+^), (d) SW620 incubated with an anti-human HLA-A2 antibody (HLA-A2^−^, MTA1^+^), (e) HT-29 (HLA-A2^−^, MTA1^+^), (f) MCF-7 (HLA-A2^+^, MTA1^+^), (g) HUVEC (HLA-A2^+^, MTA1^low^), and (h) Het-1A (HLA-A2^+^, MTA1^low^). CTLs induced by PBS were used as the negative control. Data represent means ± standard deviation (SD), ^∗^ *P* < 0.05, ^∗∗^ *P* < 0.01, and ^∗∗∗^ *P* < 0.001 vs. the PBS group.

Peptide-induced CTL lyse tumor cells by secreting cytokines such as IFN-*γ*. The IFN-*γ* secretion of CTLs induced by each peptide was detected by the ELISPOT assay. The PBMCs from six HLA-A^∗^02^+^ healthy donors were isolated to induce CTLs. As shown in Figure 2(a), the CTLs induced by each of the four peptides could produce higher levels of IFN-*γ* in the six donors (the mean spot intensities in the P22, P57, P109, and P129 groups were 566, 543, 453, and 411, respectively), with significant differences compared with the PBS control group (^∗∗∗^ *P* < 0.001).

### 3.5. *In Vitro* MTA1^+^ Tumor Cell Lysis Activity of Peptide-Specific CTLs

An LDH cytotoxicity assay was performed to assess the lysis activity of induced IFN-*γ*-producing CTLs. PBMCs from HLA-A^∗^02^+^ healthy donor no. 6 were collected to induce peptide-specific CTLs. The target cells used in this assay were the colon cancer cell lines SW620 (HLA-A^∗^0201^+^, MTA1^+^) and HT-29 (HLA-A2^−^, MTA1^+^) and the breast cancer cell line MDA-MB-231 (HLA-A^∗^0201^+^, MTA1^+^). CTLs induced by P22, P57, P109, and P129 lysed SW620 (lysis rates of 71.4%, 45.8%, 65.5%, and 38.4%, respectively) and MDA-MB-231 (lysis rates of 24.8%, 30.5%, 32.2%, and 14.1%, respectively) cells at an E/T ratio of 50 : 1 (Figures 2(b) and 2(c)). As a control, the CTLs did not lyse HT-29 (HLA-A2^−^, MTA1^+^) cells or SW620 cells incubated with an HLA-A2 blocking antibody (HLA-A2^−^, MTA1^+^) (Figures 2(d) and 2(e)). These *in vitro* ELISPOT and LDH results indicated that P22, P57, P109, and P129 can induce HLA-A2-restricted and peptide-specific CTLs to secrete IFN-*γ* and lyse target cells.

### 3.6. MTA1-Dependent Lysis of Target Cells Expressing Different Levels of MTA1

Several studies have reported that MTA1 is also expressed at a low level in most normal cells but at significantly higher levels in most cancers. We evaluated the lysis activity of MTA1-specific CD8^+^ T cells from two healthy donors toward target cells expressing different MTA1 levels. Three cell lines were used: MCF-7 cells, which expressed low levels of MTA1, and two normal human cell lines HUVEC and Het-1A. The HLA typing of these cells was HLA-A2^+^. The rates of MCF-7 cell lysis at an E/T ratio of 50 : 1 induced by P22, P57, P109, and P129 were 16.0%, 17.6%, 23.8%, and 9.34%, respectively, significantly lower than those of SW620 and MDA-MB-231 cells, and only the P57 and P109 groups have significant differences compared with the PBS control (Figure 2(f)). We found that the rates of lysis of the HUVEC and Het-1A cells by the induced CTLs were very low, with no significant differences compared with the PBS group, suggesting no significant killing of these normal cell lines (Figures 2(g) and 2(h)). Notably, the rate of Het-1A cell lysis by P109-induced CD8^+^ T cells at an E/T 50 : 1 was 11.9%; this result will require verification in more highly inducible CD8^+^ T cells. However, it is clear that the lysis rate was correlated with the MTA1 expression level in the target cells.

### 3.7. CTLs Induced by Specific Peptides in HLA-A2.1/K^b^ Transgenic Mice

**Figure 3 fig3:**
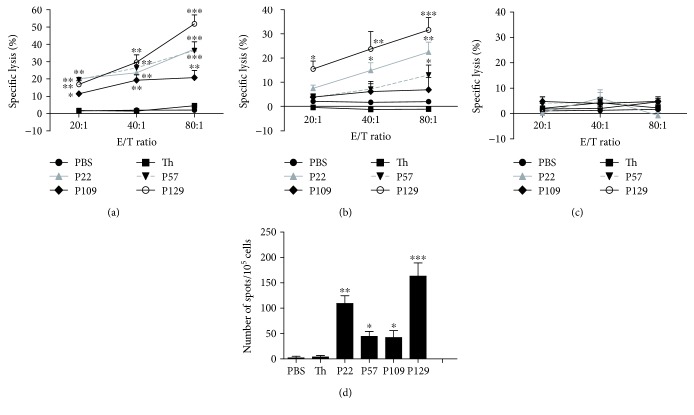
Specific lysis of target cells and secretion of IFN-*γ* by induced CTLs in immunizing HLA-A2.1/K^b^ transgenic mice immunized with the indicated peptides. Peptide (100 *μ*g) and the IA^b^-restricted HBV-core_128_ T helper epitope (140 *μ*g) emulsified in IFA were used to immunize HLA-A2.1/K^b^ transgenic mice on days 0, 5, and 10. PBS only or T helper peptide emulsified in IFA was used as the negative control. On day 11, we isolated splenocytes from the mice and restimulated the cells with 10 *μ*g/ml peptide and 10 U/ml rmIL-2 to repriming these splenocytes *in vitro* for additional 6 days. An LDH assay was performed to assess the lysis activity of the induced CTLs toward (a) SW620 cells (HLA-A2^+^, MTA1^+^), (b) T2 cells pulsed with each peptide, and (c) SW620 cells incubated with an anti-human HLA-A2 antibody (HLA-A2^−^, MTA1^+^). IFN-*γ* secretion was detected by the ELISPOT assay. (d) T2 cells incubated with each peptide and an irrelevant peptide (50 *μ*g/ml) for 4 h were used as the stimulating cells. Each sample was measured in triplicate. Data represent means ± SD (*n* = 5). ^∗^ *P* < 0.05, ^∗∗^ *P* < 0.05, and ^∗∗∗^ *P* < 0.001 vs. the T helper epitope group.

Next, we assessed whether these peptides can induce peptide-specific CTLs *in vivo* using HLA-A2.1/K^b^ transgenic mice (*n* = 5 per group). After immunizing, spleen lymphocytes were isolated and stimulated with the peptides (10 *μ*g/ml, once) for 6 days *in vitro*. Then, ELISPOT assays to measure IFN-*γ* secretion and LDH assays to evaluate target cell lysis were performed. The target cells used in this assay were SW620 colon cancer cells (HLA-A^∗^0201^+^, MTA1^+^) incubated with or without an anti-human HLA-A2 antibody and T2 cells pulsed with the corresponding peptide. The peptide-specific CTLs induced by P22 and P129 showed more potent cytotoxic activity toward SW620 cells and peptide-pulsed T2 target cells at an E/T ratio of 50 : 1 (lysis rates: 37.29% and 22.43% for P22 and 51.81% and 31.6% for P129, respectively), and differences in cytotoxicity induced by each of the four peptides versus the control group were significant (Figures 3(a)–3(c)). In addition, when SW620 cells were incubated with an anti-human HLA-A2 antibody, CTL lysis activity was no longer apparent in these cells, suggesting that these peptides induce CTL responses in peptide-specific and HLA-A2-restricted manners *in vivo*. Furthermore, the ELISPOT assay showed that P22 and P129 resulted in greater production of IFN-*γ* compared with the other peptides, and the levels of IFN-*γ* secretion induced by each of the four peptides were significantly higher compared with that of the PBS control group (Figure 3(d)).

### 3.8. *In Vitro* IFN-*γ* Secretion and Cytotoxic Activity of MTA1_(1–283)_-Induced CTLs

**Figure 4 fig4:**
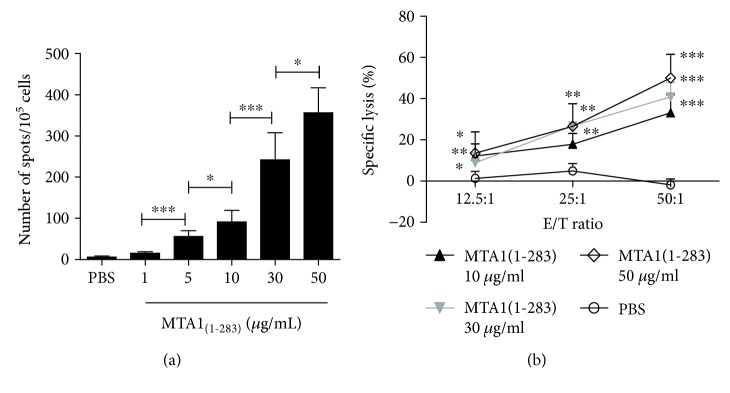
Specific T cells induced by MTA1_(1–283)_ secrete IFN-*γ* and lyse target cells. PBMCs from healthy HLA-A^∗^02^+^ donors were isolated and stimulated with protein MTA1_(1–283)_ (at final concentrations of 0, 1, 5, 10, 30, and 50 *μ*g/ml) in RPMI 1640 medium supplemented with 50 U/ml interleukin-2 and 10% FCS once a week for 21 days. On day 21, the induced T cells were collected; (a) IFN-*γ* secretion was assessed by the ELISPOT assay; ^∗^ *P* < 0.05 and ^∗∗∗^ *P* < 0.001 between two groups. (b) Cytotoxic activity was assessed by the LDH assay using SW620 cells (HLA-A2^+^, MTA1^+^) as the target cells. T cells induced by PBS were used as negative controls. ^∗^ *P* < 0.05, ^∗∗^ *P* < 0.01, and ^∗∗∗^ *P* < 0.001 vs. the control group. Data represent means ± SD (*n* = 4).

We reevaluated the epitope prediction results and found that most of the potential CTL epitopes are concentrated within the MTA1_(1–283)_ fragment. After expressing and purifying MTA1_(1–283)_, the ability of MTA1_(1–283)_ to induce CTLs in HLA-A^∗^02^+^ healthy donors was determined. In an ELISPOT assay, using T2 cells incubated with/without peptide pool (50 *μ*g/ml) as the stimulating cells, the result showed that the MTA1_(1–283)_ fragment-induced CTLs can secrete IFN-*γ* (Figure 4(a)), and ^∗^ *P* < 0.05 and ^∗∗∗^ *P* < 0.001 between two groups.

To investigate whether the CTLs induced by MTA1_(1–283)_ in HLA-A^∗^02^+^ healthy donors exhibit lysis activity, we performed an LDH assay using SW620 cells (HLA-A2^+^, MTA1^+^) as the target cells. As shown in Figure 4(b), CTLs induced by different concentrations of the MTA1_(1–283)_ fragment lysed SW620 cells, and the rate of lysis induced by 50 *μ*g/ml MTA1_(1–283)_ at an E/T ratio of 50 : 1 was 50.01%. These results suggest that the MTA1_(1–283)_ fragment induced functional CTLs to produce IFN-*γ* and kill target cells.

### 3.9. Immune Activity of MTA1_(1–283)_ in HLA-A2.1/K^b^ Transgenic Mice

**Figure 5:**
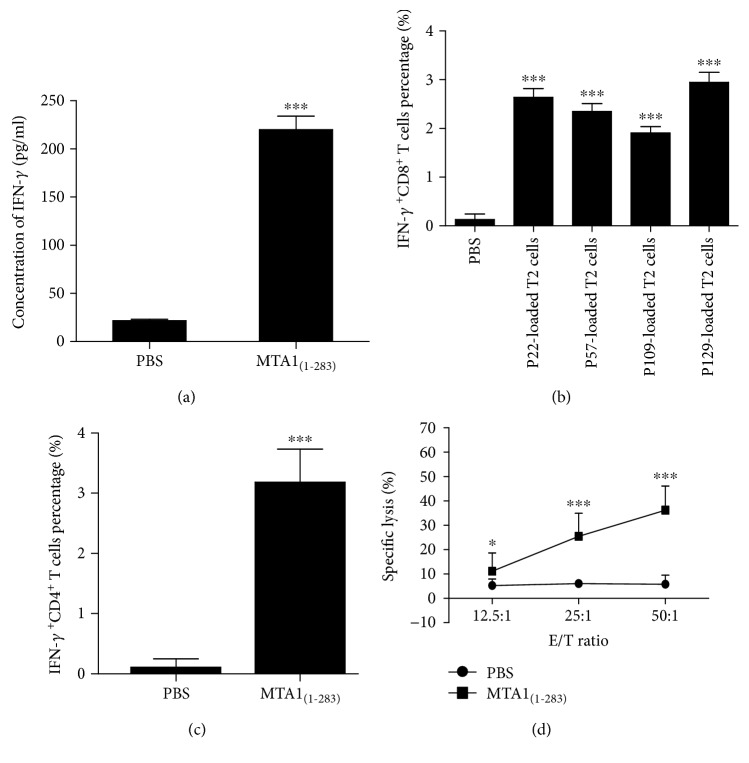
Specific lysis of various cell lines and IFN-*γ* secretion by induced T cells in HLA-A2.1/K^b^ transgenic mice immunized with MTA1_(1–283)_. MTA1_(1–283)_ (100 *μ*g/ml) or the same volume of PBS (negative control) emulsified in IFA was used to immunize HLA-A2.1/K^b^ transgenic mice on days 0 and 7. On day 14, the mice were sacrificed, and their splenocytes were collected and restimulated for another 6 days with the peptide pool (10 *μ*g/ml) and 50 U/ml rmIL-2 *in vitro*. (a) Serum was collected from the immunized mice on day 14, and the serum IFN-*γ* concentration was measured by ELISA. (b) The percentage of IFN-*γ* secreting peptide-specific CD8^+^ T cells induced by MTA1_(1–283)_ and PBS was analyzed by an intracellular staining assay. (c) The percentages of IFN-γ^+^CD4^+^ T cells in the MTA1_(1–283)_ and PBS groups were determined. (d) Specific lysis of target cells SW620 cells (HLA-A2^+^, MTA1^+^) was assessed by the LDH cytotoxicity assay. ^∗^ *P* < 0.05 and ^∗∗∗^ *P* < 0.001. *P* < 0.05 vs. the control group. Data represent means ± SD (*n* = 5).

ELISA was performed to evaluate IFN-*γ* secretion by MTA1_(1–283)_-induced CTLs *in vivo*; CTLs induced by 100 *μ*g/ml MTA1_(1–283)_ secreted more IFN-*γ* compared with the PBS control group (^∗∗∗^ *P* < 0.001) (Figure 5(a)). Furthermore, an IFN-*γ* intracellular staining assay showed that CTLs induced by 100 *μ*g/ml MTA1_(1–283)_ recognized T2 cells pulsed with each peptide and displayed a higher percentage of IFN-*γ* ^+^ CD8^+^ T cells compared with the PBS group (^∗∗∗^ *P* < 0.001) (Figure 5(b)). Subsequently, we analyzed the subsets of CD3^+^CD8^−^ cells and found that T cells induced by MTA1_(1–283)_ also consist of a certain percentage of IFN-*γ* ^+^ CD4^+^ T cells (Figure 5(c)), suggesting that MTA1_(1–283)_ contains CD4^+^ T cell epitopes that can induce IFN-*γ* secretion by CD4^+^ T cells. In the future, we plan to identify these CD4 T helper epitopes. In addition, the T cells induced by MTA1_(1–283)_ could kill SW620 tumor cells (HLA-A2^+^, MTA1^+^) (Figure 5(d)) at E/T ratios of 50 : 1 and 25 : 1, with a significant difference compared with the PBS control group (^∗∗∗^ *P* < 0.01). These results suggest MTA1_(1–283)_ can induce CTLs *in vivo* and has potential as a tumor antigen vaccine candidate.

## 4. Discussion

Tumor immunotherapy has played an important role in tumor therapy over the past 20 years. CTLs activated by derived from tumor-associated antigen HLA-restricted epitopes play a key role in specifically killing cancer cells through directly lysing tumor cells and secrete cytokines such as IFN-*γ*, TNF-*α*, and IL-2 [17, 33]. Therefore, identification of CTL epitopes from promising tumor-associated antigens (TAAs) was the key step in the development of peptide-based vaccines [34].

Tumor invasion and metastasis are the leading cause of mortality for cancer patients and are significantly associated with the expression of particular genes, such as MTA1 [35, 36]. Li et al. had identified MTA1 as a tumor-associated antigen in 2008 using the serological analysis of recombinant cDNA expression libraries (SEREX) approach [37] and found that MTA1 has a cancer-related serological profile only reacting with the serum from prostate cancer patients but not with that from any of the healthy donors tested (0/13), suggesting the presence of a natural MTA1-specific humoral immune response. Studies have shown that MTA1 is overexpressed in, and involved in the proliferation, migration, and metastasis of, a variety of tumors. But is hardly expressed in normal tissues, except for the testis [38, 39]. In addition, Plaks et al. and Feng et al. found that MTA1 maintained the stemness of cancer stem cells [35, 40]. All of the above results support MTA1 as an ideal target to identify appropriate CTL epitopes and design protein vaccines.

In this study, we found that CTL epitopes were capable of inducing tumor cell lysis by specific CTLs, suggesting that CD8^+^ T cells exhibit reactivity to MTA1 following *in vitro* peptide stimulation. We found that four peptides of MTA1, P22, P57, P109, and P129, induced CD8^+^ T cells to secrete IFN-*γ* and lyse cancer cells both *in vitro* and *in vivo*. Similar results have been reported for other tumor-associated antigens, such as survivin, PEPP2 [41], PD-L1, and the immune regulatory protein IDO [42], and natural T cell reactivity against these antigens was seen in tumor patients. In addition, we predicted the affinities of these peptides to a few of the most dominant HLA-A, -B alleles using NetCTL1.2 (Table S1), found that three peptides of MTA1, P22, P57, and P109, have certain predicted scores (threshold value of 0.75) to HLA-B8, B39, and/or HLA-B62. Furthermore, the result in Figure 5(c) suggests that MTA1_(1–283)_ contains CD4^+^ T cell epitopes that can induce IFN-*γ* secretion by CD4^+^ T cells; we predicted some 15-mers peptides that contain the predominant CTL epitopes using the online program NetMHCIIpan-3.0 (http://www.cbs.dtu.dk/services/NetMHCIIpan-3.0/) [43], which is a common pan-specific MHC class II prediction software, and found that some MHC II epitopes which have strong binders to the most frequent MHC II alleles, HLA-DRB1 [44], contain the predominant CTL epitopes of MTA1, P22, P57, P109, and P129 (Table S2). These results suggest that these peptides could have broader applicability. Several authors have reported that MTA1 is expressed at low levels in most normal cells [7, 10, 15], and accordingly, we did not detect significant lysis activity by peptide-induced CTLs toward the normal cells HUVEC and Het-1A, perhaps because of the weak MTA1 expression in these cells. Although MTA1-specific T cells can lyse MTA1^+^ tumor cells rather than normal cells, which express low levels of MTA1, and the body weight of mice in the peptide or protein group was not affected. Furthermore, like most tumor-associated antigens, MTA1 is expressed in the brain and weakly expressed in other healthy tissues, but most drugs cannot pass the blood-brain barrier; its side effects also need further consideration. In fact, MTA1 was identified by a SEREX approach, and researchers detected a natural MTA1-specific humoral immune response in prostate cancer patients but not healthy donors, indicating that MTA1 is immunogenic in patients.

MTA1 is regulated by heregulin *β*1, among other protein, and regulates target gene transcription by interacting with the NuRD complex to ultimately drive the development of malignant characteristics in tumors [45]. Recent studies found that MTA1 within the NuRD complex exists as a dimer [46, 47], and modification of MTA1 significantly affects its function. Notably, some researchers found that small molecules that interact with MTA1 destroyed the integrity of the NuRD complex and resulted in antitumor activity.

In summary, we identified four functional CTL epitopes and one epitope-enriched fragment of MTA1, MTA1_(1–283)_, and showed that these epitopes can be processed naturally. Furthermore, in the future, the identified peptides and MTA1_(1–283)_ fragment show promise for use in the design of various vaccines, including long peptide, DNA, and fusion protein vaccines, as well as in combination with immune checkpoint inhibitors.

## Figures and Tables

**Table 1:** The data of ESI–MS and HLA-A^∗^0201 binding affinity/stability properties of the peptides evaluated in this study.

Peptide	Sequence	NetCTL1.2 rank	Predicted IC50 (nM)	ESI–MS[M + H/Na]^+^		FI^a^	DC_50_^b^
				Calculated	Observed		
P22	YLIRRIEEL	1	4.9	1204.49	1204.6	2.08	>2 h
P57	ALADKHATL	5	69.6	939.4	939.5	1.56	>6 h
P109	FLSRQLESL	2	10.1	1092.1	1092.6	2.61	>6 h
P129	TLLNETESL	3	76	1019.51	1019.5	1.28	>6 h
P173	YQADITDLL	4	14.4	1051.94	1051.5	0.22	—
COX-2_321-329_	ILIGETIKI			999.6	1000.3	1.905	>6 h
HBcAg_18–27_	FLPSDFFPSV			1155.32	1155.6	1.21	>6 h

^a^FI = [mean fluorescence intensity (MFI) of the peptide − MFI background]/[MFI background]. ^b^DC_50_: time (h) required for the loss of 50% of the HLA-A^∗^0201/peptide complexes stabilized at 0 h.

## Data Availability

All the data in Table 1 and Figures 1 [Fig fig2] [Fig fig3] [Fig fig4]–5 used to support the findings of this study are available from the corresponding author upon request.

## Supplementary Materials

Supplementary Materials Figure S1: protein expression of MTA1 in human normal cell lines by Western Blot. Table S1: prediction scores of the selected four peptides of MTA1 to MHC-I supertypes. Table S2: MHC peptide binding predictions for MTA1 (the selected four peptides: P22, P57, P109, and P129) to HLA-DRB1 using NetMHCIIpan-3.0.

## References

[1] Waitz R., Solomon S. B., Petre E. N. (2012). Potent induction of tumor immunity by combining tumor cryoablation with anti-CTLA-4 therapy. *Cancer Research*.

[2] Ramamurthy C., Godwin J. L., Borghaei H. (2017). Immune checkpoint inhibitor therapy: what line of therapy and how to choose?. *Current Treatment Options in Oncology*.

[3] Gros A., Parkhurst M. R., Tran E. (2016). Prospective identification of neoantigen-specific lymphocytes in the peripheral blood of melanoma patients. *Nature Medicine*.

[4] Coulie P. G., van den Eynde B. J., van der Bruggen P., Boon T. (2014). Tumour antigens recognized by T lymphocytes: at the core of cancer immunotherapy. *Nature Reviews Cancer*.

[5] Toh Y., Pencil S. D., Nicolson G. L. (1994). A novel candidate metastasis-associated gene, mta 1, differentially expressed in highly metastatic mammary adenocarcinoma cell lines. cDNA cloning, expression, and protein analyses. *Journal of Biological Chemistry*.

[6] Reddy S. D. N., Rayala S. K., Ohshiro K. (2011). Multiple coregulatory control of tyrosine hydroxylase gene transcription. *Proceedings of the National Academy of Sciences of the United States of America*.

[7] Sen N., Gui B., Kumar R. (2014). Role of MTA1 in cancer progression and metastasis. *Cancer and Metastasis Reviews*.

[8] Dhar S., Kumar A., Gomez C. R. (2017). MTA1-activated Epi-microRNA-22 regulates E-cadherin and prostate cancer invasiveness. *FEBS Letters*.

[9] Jang K. S., Paik S. S., Chung H., Oh Y. H., Kong G. (2006). MTA1 overexpression correlates significantly with tumor grade and angiogenesis in human breast cancers. *Cancer Science*.

[10] Du B., Yang Z.-Y., Zhong X.-Y. (2011). Metastasis-associated protein 1 induces VEGF-C and facilitates lymphangiogenesis in colorectal cancer. *World Journal of Gastroenterology*.

[11] Toh Y., Oki E., Oda S. (1997). Overexpression of the MTA1 gene in gastrointestinal carcinomas: correlation with invasion and metastasis. *International Journal of Cancer*.

[12] Toh Y., Ohga T., Endo K. (2004). Expression of the metastasis-associated MTA1 protein and its relationship to deacetylation of the histone H4 in esophageal squamous cell carcinomas. *International Journal of Cancer*.

[13] Andishehtadbir A., Najvani A. D., Pardis S. (2015). Metastasis-associated protein 1 expression in oral squamous cell carcinomas: correlation with metastasis and angiogenesis. *Turkish Journal of Pathology*.

[14] Deng L., Yang H., Tang J. (2015). Inhibition of MTA1 by ER*α* contributes to protection hepatocellular carcinoma from tumor proliferation and metastasis. *Journal of Experimental & Clinical Cancer Research*.

[15] Pavlidis E. T., Pavlidis T. E. (2018). Current molecular and genetic aspects of pancreatic cancer, the role of metastasis associated proteins (MTA): a review. *Journal of Investigative Surgery*.

[16] Oany A. R., Pervin T., Mia M. (2017). Vaccinomics approach for designing potential peptide vaccine by targeting *Shigella* spp. serine protease autotransporter subfamily protein SigA. *Journal of Immunology Research*.

[17] Wu Y. H., Gao Y. F., He Y. J. (2012). A novel cytotoxic T lymphocyte epitope analogue with enhanced activity derived from cyclooxygenase-2. *Scandinavian Journal of Immunology*.

[18] Zhang X. M., Huang Y., Li Z. S., Lin H., Sui Y. F. (2010). Prediction and analysis of HLA-A2/A24-restricted cytotoxic T-lymphocyte epitopes of the tumor antigen MAGE-n using the artificial neural networks method on NetCTL1.2 server. *Oncology Letters*.

[19] Milich D. R., Hughes J. L., McLachlan A., Thornton G. B., Moriarty A. (1988). Hepatitis B synthetic immunogen comprised of nucleocapsid T-cell sites and an envelope B-cell epitope. *Proceedings of the National Academy of Sciences of the United States of America*.

[20] Shi R. R., Liu J., Zou Z. (2013). The immunogenicity of a novel cytotoxic T lymphocyte epitope from tumor antigen PL2L60 could be enhanced by 4-chlorophenylalanine substitution at position 1. *Cancer Immunology, Immunotherapy*.

[21] Hui X., Chen H., Zhang S., Ma X., Wang X., Huang B. (2011). Antitumor activities of recombinant human interferon (IFN)-*λ*1 *in vitro* and in xenograft models *in vivo* for colon cancer. *Cancer Letters*.

[22] Nijman H. W., Houbiers J. G. A., Vierboom M. P. M. (1993). Identification of peptide sequences that potentially trigger HLA-A2.1-restricted cytotoxic T lymphocytes. *European Journal of Immunology*.

[23] Gao Y. F., Sun Z. Q., Qi F. (2009). Identification of a new broad-spectrum CD8+ T cell epitope from over-expressed antigen COX-2 in esophageal carcinoma. *Cancer Letters*.

[24] Li F., Yang D., Wang Y. (2009). Identification and modification of an HLA-A^∗^0201-restricted cytotoxic T lymphocyte epitope from Ran antigen. *Cancer Immunology, Immunotherapy*.

[25] Tourdot S., Scardino A., Saloustrou E. (2000). A general strategy to enhance immunogenicity of low-affinity HLA-A2.1-associated peptides: implication in the identification of cryptic tumor epitopes. *European Journal of Immunology*.

[26] Lu Y., Ouyang K., Fang J. (2009). Improved efficacy of DNA vaccination against prostate carcinoma by boosting with recombinant protein vaccine and by introduction of a novel adjuvant epitope. *Vaccine*.

[27] Xu W., Yan M., Sun L., Zheng Z., Liu X. (2003). Ref-1 protein enhances the IL-2-stimulated telomerase activity. *Journal of Cellular Biochemistry*.

[28] Xin L., Zhang H., Zhang R. (2015). CgIL17-5, an ancient inflammatory cytokine in *Crassostrea gigas* exhibiting the heterogeneity functions compared with vertebrate interleukin17 molecules. *Developmental & Comparative Immunology*.

[29] Wu Y., Zhai W., Sun M. (2017). A novel recombinant multi-epitope vaccine could induce specific cytotoxic T lymphocyte response in vitro and in vivo. *Protein and Peptide Letters*.

[30] Liu W., Zhai M., Wu Z. (2012). Identification of a novel HLA-A2-restricted cytotoxic T lymphocyte epitope from cancer-testis antigen PLAC1 in breast cancer. *Amino Acids*.

[31] Wu Z. Y., Gao Y. F., Wu Y. H. (2011). Identification of a novel CD8+ T cell epitope derived from cancer-testis antigen MAGE-4 in oesophageal carcinoma. *Scandinavian Journal of Immunology*.

[32] Yan Z., Wu Y., du J. (2016). A novel peptide targeting Clec9a on dendritic cell for cancer immunotherapy. *Oncotarget*.

[33] Fikes J. D., Sette A. (2003). Design of multi-epitope, analogue-based cancer vaccines. *Expert Opinion on Biological Therapy*.

[34] Kumai T., Fan A., Harabuchi Y., Celis E. (2017). Cancer immunotherapy: moving forward with peptide T cell vaccines. *Current Opinion in Immunology*.

[35] Plaks V., Kong N., Werb Z. (2015). The cancer stem cell niche: how essential is the niche in regulating stemness of tumor cells?. *Cell Stem Cell*.

[36] Hanahan D., Weinberg R. A. (2011). Hallmarks of cancer: the next generation. *Cell*.

[37] Li G., Assudani D. P., Line A. (2008). Identification of metastasis associated antigen 1 (MTA1) by serological screening of prostate cancer cDNA libraries. *The Open Biochemistry Journal*.

[38] Watson P. J., Millard C. J., Riley A. M. (2016). Insights into the activation mechanism of class I HDAC complexes by inositol phosphates. *Nature Communications*.

[39] Zhao L., Niu F., Shen H., Liu X., Chen L., Niu Y. (2016). Androgen receptor and metastasis-associated protein-1 are frequently expressed in estrogen receptor negative/HER2 positive breast cancer. *Virchows Archiv*.

[40] Feng X., Zhang Q., Xia S. (2014). MTA1 overexpression induces cisplatin resistance in nasopharyngeal carcinoma by promoting cancer stem cells properties. *Molecules and Cells*.

[41] Matsushita M., Otsuka Y., Tsutsumida N. (2016). Identification of novel HLA-A^∗^24:02-restricted epitope derived from a homeobox protein expressed in hematological malignancies. *PLoS One*.

[42] Munir S., Andersen G. H., Met O. (2013). HLA-restricted CTL that are specific for the immune checkpoint ligand PD-L1 occur with high frequency in cancer patients. *Cancer Research*.

[43] Karosiene E., Rasmussen M., Blicher T., Lund O., Buus S., Nielsen M. (2013). *NetMHCIIpan-3.0*, a common pan-specific MHC class II prediction method including all three human MHC class II isotypes, HLA-DR, HLA-DP and HLA-DQ. *Immunogenetics*.

[44] Salvat R., Moise L., Bailey-Kellogg C., Griswold K. E. (2014). A high throughput MHC II binding assay for quantitative analysis of peptide epitopes. *Journal of Visualized Experiments*.

[45] Malisetty V. L., Penugurti V., Panta P., Chitta S. K., Manavathi B. (2017). MTA1 expression in human cancers—clinical and pharmacological significance. *Biomedicine & Pharmacotherapy*.

[46] Kumar R., Wang R. A. (2016). Structure, expression and functions of MTA genes. *Gene*.

[47] Millard C. J., Varma N., Saleh A. (2016). The structure of the core NuRD repression complex provides insights into its interaction with chromatin. *eLife*.

