# Supplementary material for: HLA-A2-Restricted Epitopes Identified from MTA1 Could Elicit Antigen-Specific Cytotoxic T Lymphocyte Response
Source: J Immunol Res. 2018 Nov 25;2018:2942679. doi: 10.1155/2018/2942679 (PMC6286779; doi:10.1155/2018/2942679)
Supplement: Supplementary Materials — Figure S1: protein expression of MTA1 in human normal cell lines by Western Blot. Table S1: prediction scores of the selected four peptides of MTA1 to MHC-I supertypes. Table S2: MHC peptide binding predictions for MTA1 (the selected four peptides: P22, P57, P109, and P129) to HLA-DRB1 using NetMHCIIpan-3.0. [file 2942679.f1.pdf]

# Supplementary Materials

## SUPPLEMENTARY METHODS

**Western blot assay.** The protein expression of MTA1 (81 kDa) was evaluated in two normal human cell lines HUVEC and Het-1A and human breast cancer cell lines MCF-7. In brief, The cells were collected and lysed using RIPA buffer (APPLYGEN, China);  the protein concentrations of the resulting lysates were normalized, and 30 μg protein from each cell were added to different lanes of the SDS-PAGE gel (10%) and transferred to the PVDF membrane (BIO-RAD, USA). Membranes were blocked with 5% skim milk in TBST buffer (50 mM Tris–HCl, 0.1% Tween-20, pH7.4) for about 1.5 h. Then wash the membranes three times for 5min with TBST. Further, the membranes were incubated overnight at 4℃ with the indicated primary antibody: Rabbit Anti-MTA1 antibody (Cat:5646, Dilutions: 1:500, Cell Signaling technology, USA), or Rabbit polyclonal anti-β-actin antibody (Cat: bs-0061R, Dilutions: 1:50000, Bioss Antibodies, China), and then,washed three times in TBST. Finally, corresponding secondary antibodies for MTA1 and β-actin were further incubated and the bands were visualized with the ECL plus system. Then, Gray analysis of the MTA1 protein band intensities (relative to the β-actin band intensity) was carried out by Image J.

**Predicted peptide of MTA1 to MHC-I supertypes**. we predicted the affinities of the selected four peptides to a few of the most dominant HLA-A, -B, and -C alleles using NetCTL1.2 (http://www.cbs.dtu.dk/services/NetCTL/), such as HLA-A1, A3, B7, B62, and so on. Threshold value was 0.75.

**Predicted peptide of MTA1 to the most frequent MHC II alleles**. We use the online program NetMHCIIpan-3.0 (http://www.cbs.dtu.dk/services/NetMHCIIpan-3.0/), which is commonly used to predict potential MHC II epitopes to predicted the binding affinities of some 15-mers peptides which contained these peptides to the most frequent MHC II alleles, such as

HLA-DRB1. Peptides with $IC_{50}$ below 500 nM are defined as weak binders; those with $IC_{50}$ below 50 nM as strong binders.

## SUPPLEMENTARY FIGURE LEGENDS

**Fig. S1 Protein expression of MTA1 in human normal cell lines by Western blot.**

The protein expression of MTA1 (81 kDa) was evaluated in two normal human cell lines HUVEC and Het-1A and human breast cancer cell lines MCF-7. β-actin (42 kDa) was used as loading control, and Rabbit monoconal Anti-MTA1 antibody (Cat:5646, Dilutions: 1:500, Cell Signaling technology, USA), or Rabbit polyclonal anti-β-actin antibody (Cat: bs-0061R, Dilutions: 1:50000, Bioss Antibodies, China) was used in this assay. **a)** The bands representing MTA1 and β-actin in each cancer cell line. **b)** Gray analysis of the MTA1 protein band intensities (relative to the β-actin band intensity) was carried out by Image J. **c)** Relative expression of MTA1 in different cells (relative to MCF-7 cell).

## SUPPLEMENTARY TABLES

**Table S1:** Prediction scores of the selected four peptide of MTA1 to MHC-I supertypes.

**Table S2**. MHC peptide binding predictions for MTA1 (the selected four peptide, P22, P57, P109 and P129) to HLA-DRB1 using NetMHCIIpan-3.0.

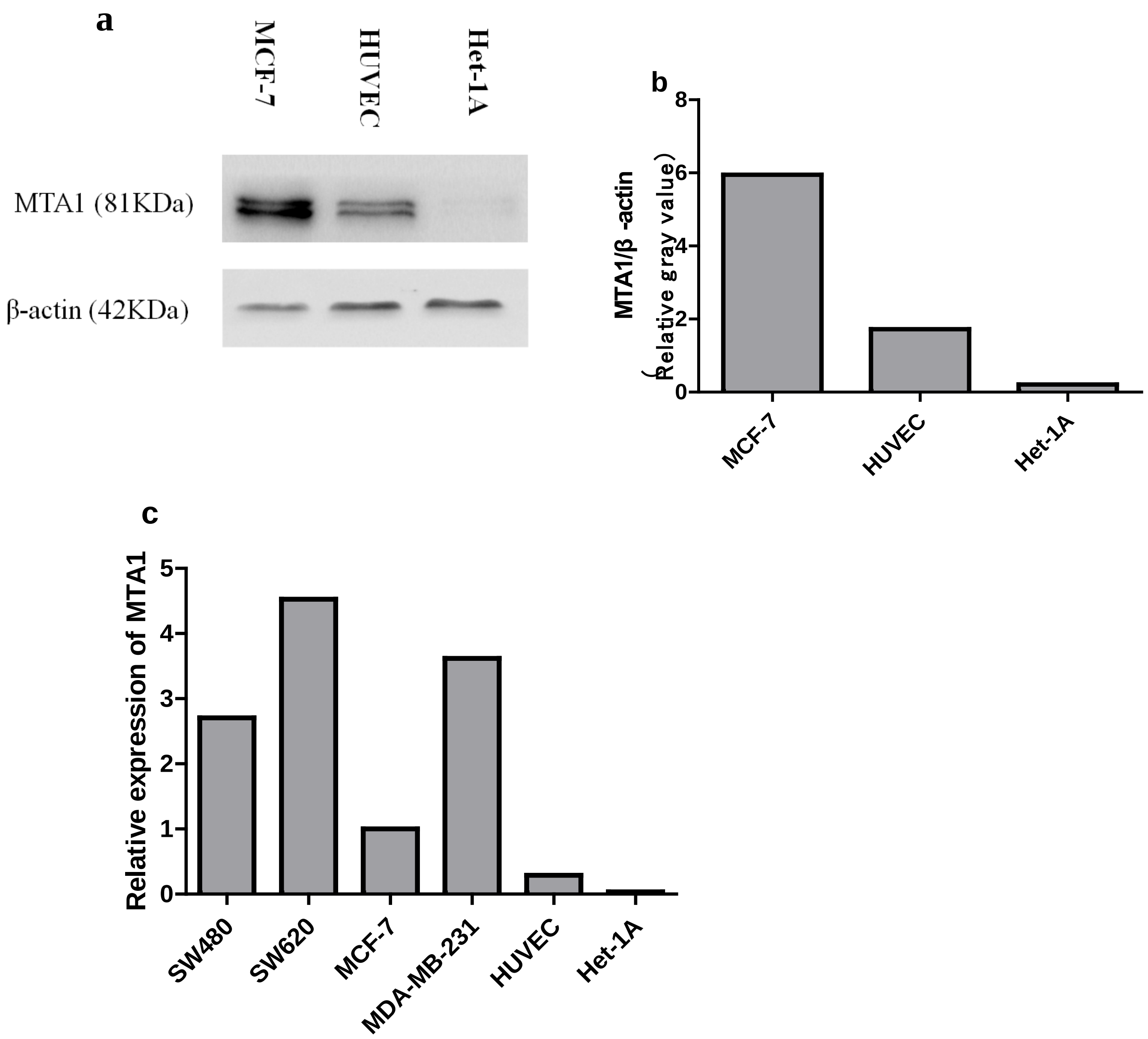


**S1 Fig. Protein expression of MTA1 in human normal cell lines by Western blot.**

The protein expression of MTA1 (81 kDa) was evaluated in two normal human cell lines HUVEC and Het-1A and human breast cancer cell lines MCF-7. β-actin (42 kDa) was used as loading control, and Rabbit monoconal Anti-MTA1 antibody (Cat:5646, Dilutions: 1:500, Cell Signaling technology, USA), or Rabbit polyclonal anti-β-actin antibody (Cat: bs-0061R, Dilutions: 1:50000, Bioss Antibodies, China) was used in this assay. **a)** The bands representing MTA1 and β-actin in each cancer cell line. **b)** Gray analysis of the MTA1 protein band intensities (relative to the β-actin band intensity) was carried out by Image J. **c)** Relative expression of MTA1 in different cells (relative to MCF-7 cell).

**Table S1** Prediction scores of the selected four peptide of MTA1 to MHC-I supertypes

| Peptide | MHC supertypes | | | | | | | | | | | |
|---|---|---|---|---|---|---|---|---|---|---|---|---|
| | HLA-A1 | HLA-A2 | HLA-A3 | HLA-A24 | HLA-A26 | HLA-B7 | HLA-B8 | HLA-B27 | HLA-B39 | HLA-B44 | HLA-B58 | HLA-B62 |
| P22 | 0.5445 | **1.3877** | 0.2231 | 0.3842 | **0.8802** | 0.3493 | **2.1825** | 0.3338 | **0.8917** | 0.2863 | 0.4467 | **1.0868** |
| P57 | 0.5384 | **1.1876** | 0.2884 | 0.3344 | 0.4121 | 0.6123 | **0.7919** | 0.3405 | **1.1256** | 0.3003 | 0.2944 | **0.8449** |
| P109 | 0.4941 | **1.3183** | 0.2402 | 0.3060 | 0.3831 | 0.3794 | **1.5234** | 0.3006 | **0.8443** | 0.2912 | 0.3871 | 0.6201 |
| P129 | 0.4781 | **1.2249** | 0.2465 | 0.3050 | 0.3833 | 0.3702 | 0.4467 | 0.3019 | 0.6924 | 0.2796 | 0.2972 | 0.5787 |

Threshold value: 0.75

Bold display with a threshold > 0.75

**Table S2** MHC peptide binding predictions for MTA1 (the selected four peptide, P22, P57, P109 and P129) to HLA-DRB1 using NetMHCIIpan-3.0

| Selected Peptide | sequence | position | $IC_{50}$(nM) | | | | | |
|---|---|---|---|---|---|---|---|---|
| | | | HLA-DRB1*0101 | HLA-DRB1*0301 | HLA-DRB1*0401 | HLA-DRB1*0701 | HLA-DRB1*1101 | HLA-DRB1*1501 |
| P22 | SSNPYLIRRIEELNK | 18-32 | 349.34 | - | - | 494.20 | 412.21 | - |
| | SNPYLIRRIEELNKT | 19-33 | 255.49 | - | - | 467.65 | 354.80 | - |
| | NPYLIRRIEELNKTA | 20-34 | 302.54 | - | - | - | 345.61 | - |
| | PYLIRRIEELNKTAN | 21-35 | 354.73 | - | - | - | 369.15 | - |
| | YLIRRIEELNKTANG | 22.36 | 482.47 | - | - | - | - | - |
| P57 | ISSTLIALADKHATL | 51-65 | **19.93** | 398.78 | 148.36 | 212.20 | 137.81 | 171.25 |
| | SSTLIALADKHATLS | 52-66 | **17.47** | 315.59 | 137.85 | 265.80 | 133.10 | 170.01 |
| | STLIALADKHATLSV | 53-67 | **25.31** | 241.79 | 199.77 | 289.44 | 151.65 | 226.23 |
| | TLIALADKHATLSVC | 54-68 | **40.18** | 282.98 | 320.19 | 365.28 | 221.22 | 331.14 |
| | LIALADKHATLSVCY | 55-69 | 83.07 | 487.48 | - | 487.58 | - | - |
| | IALADKHATLSVCYK | 56-70 | 463.94 | - | - | - | - | - |
| | ALADKHATLSVCYKA | 57-71 | 494.13 | - | - | - | - | - |

| | | | | | | | | |
|---|---|---|---|---|---|---|---|---|
| P109 | LRHRELFLSRQLESL | 103-117 | 143.33 | - | - | 203.71 | 152.87 | 316.62 |
| | RHRELFLSRQLESLP | 104-118 | 159.58 | - | - | 238.08 | 229.58 | 461.10 |
| | HRELFLSRQLESLPA | 105-119 | 78.12 | - | - | 216.26 | 250.02 | 440.75 |
| | RELFLSRQLESLPAT | 106-120 | **46.82** | - | 408.39 | 196.75 | 265.31 | 443.91 |
| | ELFLSRQLESLPATH | 107-121 | **37.55** | - | 302.65 | 247.48 | 417.71 | - |
| | LFLSRQLESLPATHI | 108-122 | **10.48** | - | 121.58 | 52.88 | 334.16 | 243.79 |
| | FLSRQLESLPATHIR | 109-123 | **7.61** | - | 94.54 | **36.25** | 320.13 | 180.37 |
| P129 | KCSVTLLNETESLKS | 125-139 | 450.52 | - | - | - | - | - |
| | CSVTLLNETESLKSY | 126-140 | 329.19 | - | - | - | - | - |
| | SVTLLNETESLKSYL | 127-141 | 317.66 | 414.19 | - | - | - | - |
| | VTLLNETESLKSYLE | 128-142 | 398.56 | - | - | - | - | - |

Peptides with $IC_{50}$ below 500 nM are defined as weak binders; those with $IC_{50}$ below 50 nM as strong binders, and IC50 (nM) < 50 display with Bold font, $IC_{50}$ (nM) > 500 do not display.
